# Radiological Findings of Prostatic Arterial Anatomy for Prostatic Arterial Embolization: Preliminary Study in 55 Chinese Patients with Benign Prostatic Hyperplasia

**DOI:** 10.1371/journal.pone.0132678

**Published:** 2015-07-20

**Authors:** Guodong Zhang, Maoqiang Wang, Feng Duan, Kai Yuan, Kai Li, Jieyu Yan, Zhongfei Chang, Yan Wang

**Affiliations:** Department of Interventional Radiology, Chinese PLA General Hospital, Beijing, China; University of Washington School of Medicine, UNITED STATES

## Abstract

**Objective:**

To describe the prostatic arterial supply using Cone-beam computed tomography (CT) and digital subtraction angiography (DSA) before prostatic arterial embolization (PAE) for benign prostatic hyperplasia (BPH).

**Methods:**

In a retrospective study from January 2012 to January 2014, 55 male patients (110 hemipelves) with BPH who underwent PAE were evaluated by Cone-beam CT in addition to pelvic DSA during embolization planning. Each hemipelvis was evaluated regarding the number of prostatic arteries (PA) and their origins, diameters, territorial perfusion, and anastomoses with adjacent arteries.

**Results:**

A total of 114 PAs were identified in 110 hemipelves. There was one PA in 96.4% of the hemipelves (n=106), and two independent PAs in the other 3.6% (n=4). The PA was found to originate from the anterior trunk of the internal iliac artery in 39.5% of cases (n=45) , from the superior vesical artery in 32.6% (n=37), and from the internal pudendal artery in 27.9% of cases (n=32). Extra-prostatic anastomoses between PA and adjacent arteries were found in 39.1% of hemipelves (n=43). Intra-prostatic anastomoses between PAs and contra-lateral prostatic branches were found in 61.8% of hemipelves (n=68). In 67.3% of our study population (n=37), the prostate was dominantly supplied via a unilateral PA.

**Conclusion:**

The prostatic vascularization is complex with frequent anatomic variations. Knowledge of the vascular anatomy of the prostate may provide indications for planning PAE and avoiding nontarget embolization.

## Introduction

Prostatic arterial embolization (PAE) is a minimally invasive alternative treatment for moderate- or severe-grade lower urinary tract symptoms (LUTS) secondary to benign prostatic hyperplasia (BPH). Clinical outcomes such as symptom improvement with reduction of the prostate size have been reported in short- and intermediate-term studies [1–3].

Because the arterial supply to the prostate is highly variable, the great challenge during PAE is to differentiate the prostatic arteries (PAs) from adjacent arteries and detect small arterial anatomoses. Previous cadaveric and radiological anatomic descriptions of PAs were hampered by lack of consistency [4–9]. Furthermore, intraprocedural Cone-beam computed tomography (CT) was absent in the study addressing detailed anatomy of prostatic arterial anastomoses and origins [7,8]. Cone-beam CT has the ability to provide three-dimensional vascular and more subtle vascular information compared with conventional DSA [10–14]. In the present study, Cone-beam CT was incorporated into the PAE planning. The evaluation was based on a retrospective analysis of DSA and Cone-beam CT of 55 patients with BPH. Our study is intended as a supplement to the previous studies investigating the prostatic arterial supply.

## Methods

### Ethics Statement

The study complies with current ethical consideration and was approved by the institutional review board of Chinese People’s Liberation Army General Hospital. The procedures were explained in full to each patient, and written informed consent for PAE was obtained from all patients.

### Patient Selection

From January 2012 to January 2014, 64 male patients with moderate- or severe-grade LUTS related to BPH underwent selective pelvic DSA and Cone-beam CT before PAE.

Pre-operative assessment included prostate volume measurement by pelvic magnetic resonance imaging (MRI), serum prostate-specific antigen(PSA) measurement, international prostate symptom score(IPSS), quality of life assessment(QoL), peak urinary flow rate, international index of erectile function score(IIEF). We performed prostatic biopsy in the cases of suspicious prostatic malignancy. Patients with proven prostatic malignancy, large bladder diverticula, renal insufficiency, active urinary infection, neurogenic bladder, unregulated coagulation parameter or contrast agent allergy were excluded. Only the patients who underwent successful bilateral PA catheterization were included in our study.

### Equipment and procedures

In our study, patients underwent DSA and PAE in the Cone-beam CT-capable uniplanar flat-panel interventional suite (Innova 4100-IQ; GE, USA). Intervention was performed under local anesthesia through a unilateral transfemoral access in all patients with the use of nonionic contrast medium (Iodixanol, 320 mg I/ml; GE Healthcare, USA). A 4-Fr Simmons I catheter (Cordis, USA) was inserted through the sheath placed in the femoral artery and was formed at the level of the aortic arch. The Simmons catheter was then pulled back to selective catheterize the contralateral hypogastric artery and its anterior division negotiating with a hydrophilic guide-wire (Terumo, Tokyo, Japan). The DSA was performed with 15 mL of nonionic contrast medium at a flow rate of 5 mL/s with the anterior—posterior view and ipsilateral anterior oblique 30° view. Subsequently, Cone-beam CT was performed at a delay time 5 sec after automatic injection of 24 mL contrast medium at a flow rate of 4 mL/s.

All the PAs identified in pelvic DSA were catheterized using a 2.8Fr micro-catheter (Progreat; Terumo, Tokyo, Japan) and a 0.018- inch micro guide-wire (Terumo, Tokyo, Japan) with the use of roadmap guidance. Initial PA angiography was routinely performed by manually to ensure that the tip of the microcatheter and to evaluate the blood supply to the prostate. The microcatheter tip was located in the proximal part of the middle third of PA. The subsequent DSA and Cone-beam CT were performed with 4–8 mL contrast medium by injecting over a period of 3–4 seconds and an imaging delay of 3–5 seconds. The injection pressure on 2.8F catheter was set at 300–500 psi (1 psi is equal to 6.895 kPa). Injection rates and contrast medium volumes varied according to size of the PAs. The Cone-beam CT imaging sequence used an 18 sec rotational scan of 180° at a rotation speed of 10° per second. The volumetric data sets were automatically transferred to a separate post-processing workstation (Advantage Workstation4.3; GE Healthcare) for three-dimensional reconstruction.

To confirm the prostatic blood supply and avoid potential nontarget embolization, selective DSA and Cone-beam CT was also performed in the questionable artery with an uncertain tissue perfusion. We altered the catheter reposition in response to the Cone-beam CT acquisitions. PAE was performed with 90-180-μm nonspherical polyvinyl foam embolic particles (Cook Medical, USA). After embolization of the contralateral prostatic artery was completed, the other side prostatic artery was embolized with the same technique.

The imaging acquisitions of DSA combined with Cone-beam CT were assessed by two interventional radiologists with more than 10 years of experience. The number, origins, trajectory, pattern, diameter (The diameter of the artery, relative to that of the nearby catheter tip, was measured on magnified images and was calculated as arterial size divided by catheter size multiplied by the actual diameter of the micro-catheter used), and anastomoses of PA were assessed. The contrast-enhanced territories shown by PA Cone-beam CT with selective intra-arterial contrast agent administration were used to reveal territories vascularized by the catheterized PA and predict the distribution of embolic particles. The stained area was estimated on axial images of contrast-enhanced Cone-beam CT. After summation of the stained area, we calculated the percentage of perfused prostate in relation to the whole prostate. In addition, the percentage of perfused prostate in relation to the whole prostate as seen on Cone-beam CT was used as an adjunct to DSA for assessing the proportion of prostatic arterial supply via each side PA. We defined that prostate was dominantly vascularized by unilateral PA when the proportion of prostatic arterial supply via one side PA was over 70%.

### Statistical analysis

Descriptive statistics were used to present results as absolute numbers (n) and percentages(%) of the total study population.

## Results

### Technical results

During our study period, 64 consecutive patients underwent selective DSA and Cone-beam CT. 5 patients received only unilateral PA catheterization and 1 patient did not receive selective PA catheterization on either hemipelves due to the atherosclerotic occlusion of the prostatic arteries. In addition, the Cone-beam CT imaging could not be interpreted due to technical problem in 3 patients. A total of 9 patients were excluded according to the study protocol.

A total of 55 patients who underwent successful bilateral DSA and Cone-beam CT were included for evaluation in our study. The included patient age ranged from 52 to 84 years, with a mean of 65.1 years. We altered the catheter placement in response to the Cone-beam CT acquisitions until we had confidence before embolization. A total of 43 patients received bilateral embolization and the other 12 patients received unilateral embolization because sites of potential nontarget embolization were still identified on CBCT after further alteration of the microcatheter placement in 11 patients and a technical obstacle happened during embolization in 1 patient.

### PA numbers and origins

Imaging acquisitions confirmed a total of 114 PAs arising from 110 internal iliac arteries, including one independent PA in 96.4% of hemipelves (n = 106) and two independent PAs in the other 3.6% (n = 4).

**Fig 1:**
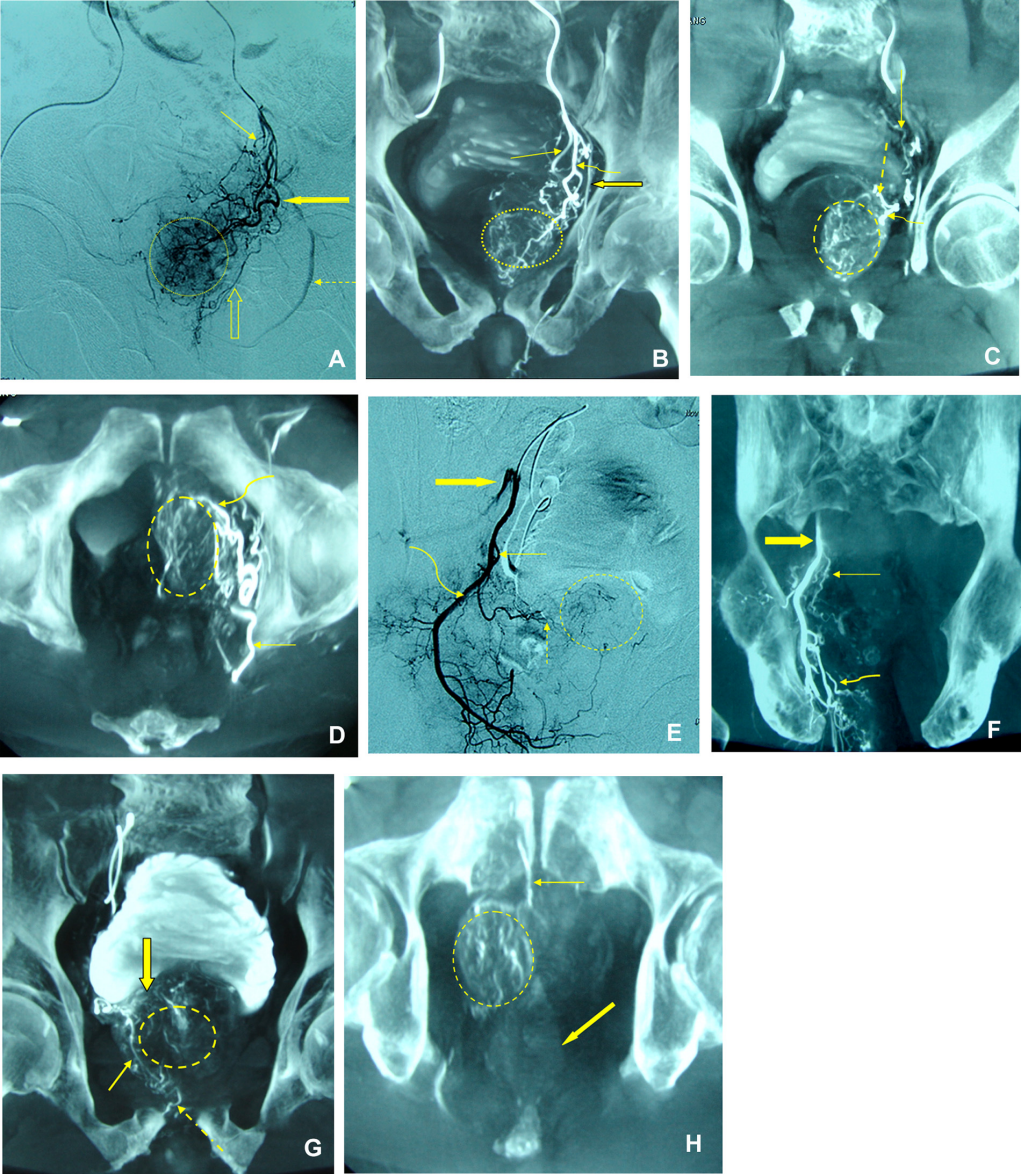
Images from a 71-year-old male patient with benign prostatic hyperplasia. (a). DSA of the anterior division of left internal iliac artery performed with same-side anterior oblique projection (30°). The straight arrow marks the superior vesical artery, the thick arrow marks the left prostatic artery (PA), dotted arrow marks the internal pudendal artery, and the open arrow marks the accessory pudendal artery. Prostate gland opacification is seen inside the dotted circle. (b). Coronal Cone-beam CT performed after selective catheterization of the anterior division of left internal iliac artery. The curved arrow marks the left PA, the straight arrow marks the superior vesical artery, and the thick arrow marks the internal pudendal artery. The prostate gland perfusion is seen inside the dotted circle. The left PA originates from the anterior common gluteal-pudendal trunk. (c). Coronal Cone-beam CT performed after selective catheterization of the left PA. The PA bifurcates into anterior/lateral (curved arrow) and inferior vesical artery (dotted arrow), and the straight arrow marks the superior vesical artery. The prostate gland perfusion is seen inside the dotted circle. The image showed that the left PA vascularizing majority of the prostate gland. (d). Axial cone-beam CT performed after selective catheterization of the left PA (straight arrow). The curved arrow marks the anterior/lateral PA. The prostate gland perfusion is seen inside the dotted circle. (e). DSA of the anterior division of right internal iliac artery performed with same-side anterior oblique projection (30°). The straight arrow marks the right PA, the dotted arrow marks the anterior/lateral branch of the right PA, the curved arrow marks the internal pudendal artery, and the thick arrow marks the inferior gluteal artery. Prostate gland opacification is seen inside the dotted circle. (f). Coronal Cone-beam CT performed after selective catheterization of the anterior division of right internal iliac artery. The straight arrow marks the right PA had a superior origin from the internal pudendal artery (thick arrow), the curved arrow marks the rectal artery. The right PA originates from the internal pudendal artery. (g). Coronal Cone-beam CT performed after selective catheterization of the right PA. The PA bifurcates into anterior/lateral (straight arrow) and posterior/lateral PAs (thick arrow) with anastomoses to the internal pudendal artery (dotted arrow) rendering potential nontarget embolization with small-sized embolic particles. The prostate gland perfusion is seen inside the dotted circle. The imaging showed that the right PA vascularized minority of the prostate gland. (h). Axial Cone-beam CT performed after selective catheterization of the right PA. The straight arrow marks the PA anastomoses to the internal pudendal artery, and the thick arrow marks the rectum. The prostate gland perfusion is seen inside the dotted circle. In this case, anastomosis occlusion with micro-coils prior to PAE is a better option.

PAs had variable origins between the left and right sides. The left and right PA origins were asymmetric in 65.5% of patients (n = 36) and symmetric in the other 34.5% of patients (n = 19). The most frequent origin was the gluteal-pudendal trunk (39.5%; n = 45) (Fig 1), the superior vesical artery (32.6%; n = 37) (Figure A and Figure B in S1 Folder), and the internal pudendal artery (27.9%; n = 32) (Fig 1). So a total 72.1% of PAs had a superior origin from the gluteal-pudendal trunk or the superior vesical artery, and 27.9% had an inferior origin from the internal pudendal artery, above the sciatic notch (listed in S1 Table).

### Diameter and pattern of PA

The microcatheter tip was located in the proximal part of the middle third of PA when the PA diameter was measured. This point was usually where we performed embolization. Diameter and pattern of PA were variable: Mean diameter of PA was 0.9mm (range, 0.5–1.5mm). The PA with a corkscrew pattern of the capsular branches was found in 30.9% of individuals (n = 17).

### PA anastomosis

Cone-beam CT and DSA were able to portray the intraprostatic branches and PA anastomoses with adjacent arteries. Extra-prostatic anastomoses between PAs and adjacent arteries were identified in 39.1% (43/110) of hemipelves. The most frequent anastomosis types found were to the internal pudendal artery (25.5%, 28/110), the rectal branches(16.4%, 18/110),both the internal pudendal artery and rectal branches(6.4%, 7/110), and the inferior vesical artery(3.6%, 4/110). Prostatic arteries were found to have no significant anastomoses to the obturator artery or the inferior gluteal artery. In addition, intra-prostatic anastomoses between PAs and contra-lateral prostatic branches were found in 61.8% of hemipelves (n = 68)(listed in S2 Table).

### Intra-prostatic arterial supply proportion

The proportion of prostatic arterial supply was variable between via the left and right side PA. In 20 of 55 patients, the left side PA provided over 70% of the arterial supply of prostatic gland. In another 17 of 55 patients, the right side PA provided over 70% of the arterial supply of prostatic gland. So in a total 67.3% of 55 patients (n = 37), a unilateral PA vascularizing over 70% of the prostatic gland (Fig 1).

### Other finding

The amount of arterial anastomoses detected was variable depending on injection rate, volume and pressure of contrast agent used during PA angiography. Selective PA DSA performed with contrast agent manual injection showed less arterial anastomoses compared with automatic injection using contrast agent injector. These arterial anastomoses were most involved in anastomoses between PA and rectal arteies, and anastomoses between PA and penile arteries. After catheterization in rectal artery, internal pudendal artery, and inferior vesical artery, selective DSA performed with contrast agent automatic injection might show arterial branches supplying the peripheral prostate gland. Partial of these arterial anastomoses and arterial branches may be an artifact of the strength of injection and not based on the physiologic flow.

## Discussion

The descriptions of prostatic arterial anatomy based on cadaveric study have been published [4–6]. However, pelvic vascular anatomy is very difficult to evaluate in cadaveric specimens because of the almost indistinguishable fat and small nerves and vessels [15, 16]. Conventional cadaveric dissections of pelvic region can destroy the relevant structures and distort relationships. More important, in contrast to the vivo studies, most cadaveric specimens had normal-sized prostates, so it was impossible to extrapolate completely the anatomic findings in patients with BPH [6].

From our experience with PAE, we sometime could not differentiate PAs from the surrounding arterial branches by using conventional DSA alone despite angiography in multiple obliquities. And the PA origin could not be identified sometimes by using DSA alone because of superimpositions. Then in the subsequent study with PAE, we routinely incorporated Cone-beam CT into treatment planning for PAE. In our experience with PAE, by using of Cone-beam CT in anterior division of hypogastric artery, the small PAs could be differentiated from the adjacent or overlapping arteries and the PA origin was demonstrated clearly.

In the present study, the frequency of identifiable PA and anastomosis were somewhat different from previously published results. First, two independent PAs were found in 3.6% of the pelvic sides in present study, in contrast to 43% in the study by Bilhim et al. Second, a total of extra-prostatic and intra-prostatic anastomoses were identified in approximately 61% of pelvic sides in present study, in contrast to 57% in previous study. This discrepancy may be explained by distinct techniques used in these two studies and suggested that these two patients population might be different. Besides, in present study, we found that the frequency of PAs and arterial anastomoses detected was variable depending on injection rate, volume and pressure of contrast agent used. Partial of these PAs and arterial anastomoses might be an artifact of the strength of injection and not based on the physiologic flow. This was another reason for the discrepancy between our study and previous study beyond the potential for different patient populations. Finally, from our experience, Cone-beam CT used in present study identified more subtle vascular anastomoses compared with DSA.

One of the key techniques for PAE is to avoid hazardous nontarget embolization of the branches supplying the penis, bladder and rectum. Embolization to one of these nontarget sites can carry serious morbidity. Nontarget embolization is often related to the small particle size, embolization rate, non-controlled injection, incorrect identification of PA, or the presence of unrecognized arterial anastomoses. The cases of bladder wall ischemia and transient ischemic rectitis resulting from nontarget embolization had been reported [3,17]. Accurate detection of PA anastomoses is indispensable for the technical success of this procedure. In the present study, both vascular and parenchymal enhancements were sufficiently visualized on all Cone-beam CT scans with the tip of the catheter positioned in the PA. So the additional Cone-beam CT was used as an adjunctive tool to conventional DSA for evaluating the prostatic arterial supply and detecting the small anastomoses. The additional imaging information provided by Cone-beam CT enables the operator to adequately identify potential nontarget embolization sites.

Another surprising finding in present study was that in 67.3% of the individuals, prostatic gland perfusion ranges by unilateral PA was over 70 percent. We defined this prostate was dominantly vascularized via unilateral PA. And PA anastomoses with contra-lateral PAs were identified in 61% of pelvic sides in present study. These imaging findings might predict some patients underwent unilateral PAE also will have good clinical outcomes.

The disadvantages of the incorporating Cone-beam CT into the treatment planning included more intra-procedural contrast agent used and more radiation exposure to the patients. There are three limitations to the present study. First, the sample size is not enough. The 55 individuals maybe underestimate the radiological findings of the large population of China. Second, preoperative Pelvic CTA was absent, which may offer information of the tortuosity or atherosclerotic changes in arteries that is useful to preoperative planning for PAE. Third, no evaluation was performed of how the knowledge gained from routine use of Cone-beam CT reduced the complication rate in the present study.

From our experience, using the combined technique of Cone-beam CT and DSA we were able to depict radiological prostatic arterial anatomy and certify correct catheter position for PAE. The findings of prostatic arterial anatomy were helpful to achieve optimal target embolization and avoid complications from nontarget embolization.

## Supporting Information

S1 Folder Images from a 61-year-old male patient with benign prostatic hyperplasia. (A). DSA of the anterior division of left internal iliac artery performed with same-side anterior oblique projection (30°). The straight arrow marks the left prostatic artery (PA) originating from the superior vesical artery(the thick arrow). (B). Coronal Cone-beam CT performed after selective catheterization of the left PA. The left PA (the thick arrow) originates from the superior vesical artery(straight arrow).
(ZIP)

S1 Table Origin of PA. (DOC)

S2 Table Arterial anastomosis type of PA. (DOC)
