# Supplementary figures and images for: Radiological Findings of Prostatic Arterial Anatomy for Prostatic Arterial Embolization: Preliminary Study in 55 Chinese Patients with Benign Prostatic Hyperplasia
Source: PLoS One. 2015 Jul 20;10(7):e0132678. doi: 10.1371/journal.pone.0132678 (PMC4508051; doi:10.1371/journal.pone.0132678)

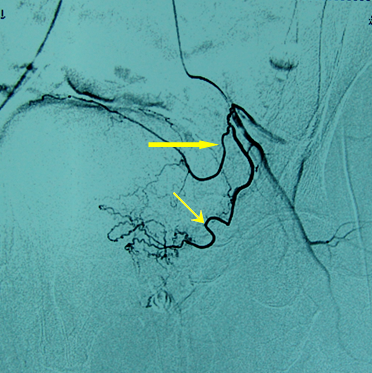

Supplement: S1 Folder — (A). DSA of the anterior division of left internal iliac artery performed with same-side anterior oblique projection (30°). The straight arrow marks the left prostatic artery (PA) originating from the superior vesical artery(the thick arrow). (B). Coronal Cone-beam CT performed after selective catheterization of the left PA. The left PA (the thick arrow) originates from the superior vesical artery(straight arrow). (ZIP) [file pone.0132678.s001.zip › Figure A.tif]

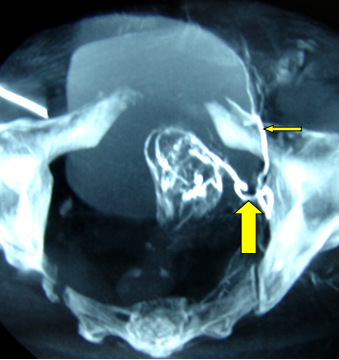

Supplement: S1 Folder — (A). DSA of the anterior division of left internal iliac artery performed with same-side anterior oblique projection (30°). The straight arrow marks the left prostatic artery (PA) originating from the superior vesical artery(the thick arrow). (B). Coronal Cone-beam CT performed after selective catheterization of the left PA. The left PA (the thick arrow) originates from the superior vesical artery(straight arrow). (ZIP) [file pone.0132678.s001.zip › Figure B.tif]
