# Supplementary material for: Radiological Findings of Prostatic Arterial Anatomy for Prostatic Arterial Embolization: Preliminary Study in 55 Chinese Patients with Benign Prostatic Hyperplasia
Source: PLoS One. 2015 Jul 20;10(7):e0132678. doi: 10.1371/journal.pone.0132678 (PMC4508051; doi:10.1371/journal.pone.0132678)
Supplement: S1 Table — (DOC) [file pone.0132678.s002.doc]

**S1 Table** . Origin of PA.

| PA arises from | n* | % |
| --- | --- | --- |
| Gluteal-pudendal trunk | 45 | 39.5% |
| Superior vesical artery | 37 | 32.6% |
| Internal pudendal artery | 32 | 27.9% |

PA = prostatic artery. *Number of arteries
