# Supplementary material for: Radiological Findings of Prostatic Arterial Anatomy for Prostatic Arterial Embolization: Preliminary Study in 55 Chinese Patients with Benign Prostatic Hyperplasia
Source: PLoS One. 2015 Jul 20;10(7):e0132678. doi: 10.1371/journal.pone.0132678 (PMC4508051; doi:10.1371/journal.pone.0132678)
Supplement: S2 Table — (DOC) [file pone.0132678.s003.doc]

**S2 Table** . Arterial anastomosis type of PA.

| PA anastomoses with | n* | % |
| --- | --- | --- |
| Internal pudendal artery | 28 | 25.5% |
| Rectal branches | 18 | 16.4% |
| Both the internal pudendal artery and rectal branches | 7 | 6.4% |
| Inferior vesical artery | 4 | 3.6% |
| Contralateral PAs | 68 | 61.8% |

PA = prostatic artery. *Number of arteries
